# Implications of Innate Immunity in Post-Acute Sequelae of Non-Persistent Viral Infections

**DOI:** 10.3390/cells10082134

**Published:** 2021-08-19

**Authors:** Maximilian Hirschenberger, Victoria Hunszinger, Konstantin Maria Johannes Sparrer

**Affiliations:** Institute of Molecular Virology, Ulm University Medical Center, 89081 Ulm, Germany; maximilian.hirschenberger@uni-ulm.de (M.H.); victoria.hunszinger@uni-ulm.de (V.H.)

**Keywords:** virus, cytokines, interferon, innate immunity, inflammation, post-acute sequelae, long COVID, PASC, long hauler syndrome, chronic, acute, post-acute

## Abstract

Non-persistent viruses classically cause transient, acute infections triggering immune responses aimed at the elimination of the pathogen. Successful viruses evolved strategies to manipulate and evade these anti-viral defenses. Symptoms during the acute phase are often linked to dysregulated immune responses that disappear once the patient recovers. In some patients, however, symptoms persist or new symptoms emerge beyond the acute phase. Conditions resulting from previous transient infection are termed post-acute sequelae (PAS) and were reported for a wide range of non-persistent viruses such as rota-, influenza- or polioviruses. Here we provide an overview of non-persistent viral pathogens reported to be associated with diverse PAS, among them chronic fatigue, auto-immune disorders, or neurological complications and highlight known mechanistic details. Recently, the emergence of post-acute sequelae of COVID-19 (PASC) or long COVID highlighted the impact of PAS. Notably, PAS of non-persistent infections often resemble symptoms of persistent viral infections, defined by chronic inflammation. Inflammation maintained after the acute phase may be a key driver of PAS of non-persistent viruses. Therefore, we explore current insights into aberrant activation of innate immune signaling pathways in the post-acute phase of non-persistent viruses. Finally, conclusions are drawn and future perspectives for treatment and prevention of PAS are discussed.

## 1. Introduction

Viral pathogens have a pronounced impact on human health worldwide. Notably, the recently emerged coronavirus disease 2019 (COVID-19) caused more than 4.1 million deaths with over 192 million cases globally (https://covid19.who.int, accessed on 23 July 2021). While some virus species like members of the Herpesvirus family establish life-long infections, the majority of the currently known human pathogenic viruses generally cause transient acute infections. The acute phase is usually accompanied by a spike in inflammatory cytokines that are released as the infection is detected by the host’s immune system and countermeasures are initiated.

### 1.1. Acute Viral Infections Trigger and Modulate the Innate Immune Responses

The human immune system can be divided into two major parts, the adaptive and the innate immune system. The primary function of the innate immune system is to sense incoming pathogens, mount rapid responses, including cytokine release, that set cells in an anti-viral state and activate and recruit the adaptive immune system [1,2]. Ultimately it plays a major role in the elimination of viral pathogens. It has the capability to be activated by specific patterns derived from invading pathogens (pathogen associated molecular patterns, PAMPS), invading commensals (microbe associated molecular patterns, MAMPs) or even endogenous signals released, e.g., upon tissue damage (danger associated molecular patterns, DAMPs). These patterns are sensed by dedicated germ-line encoded pattern recognition receptors (PRRs). For example, Toll-like-receptors are located to endosomes or the plasma membrane facing the topologically external side [3]. Intracellular PRRs like the RIG-like receptors, retinoic acid-inducible gene I (RIG-I) and melanoma differentiation-associated protein 5 (MDA5) or cyclic GMP-AMP synthase (cGAS) interact with PAMPs inside the cell [4]. Each of these receptors recognizes specific patterns, e.g., TLR4 detects bacterial lipopolysaccharides (LPS), TLR3 binds double-stranded RNA, whereas the intracellular RIG-I detects tri- or di-phosphorylated RNA in the cytoplasm and cGas senses DNA [4]. Activation of these sensors leads to the induction of signaling cascades that eventually induce a wide variety of immune responses, e.g., the induction of pro-inflammatory cytokines. The initial recognition of the pathogen at the site of infection or by dedicated innate immune cells like dendritic cells leads to transcription, translation and secretion of a first wave of cytokines. These include type I interferons (type I IFNs) and other (pro-) inflammatory cytokines. As an integral part of the innate immune response, these cytokines set cells in an anti-viral state by inducing a transcriptional program that recruits and triggers adaptive immune responses. Activated immune cells may then proceed to release a second wave (often including type II IFNs) that mitigates the damage of the first wave and further facilitates the clearance of the pathogen. To overcome the powerful anti-viral barrier of the innate immune system, successful viruses, including influenza A virus (IAV) or the severe acute respiratory coronavirus 2 (SARS-CoV-2) have evolved strategies to counteract, evade and sometimes even exploit innate immune signaling pathways to promote their replication [5,6,7]. However, the combination of triggering of innate immune system by viral pathogens and manipulation, evasion, and counteraction of these responses by the viral pathogen often leads to aberrant immune reactions. These excessive immune reactions—in contrast to their usual function—fail to eliminate the pathogen but contribute to the severity of the disease. One major example is cytokine storms, which are somewhat loosely defined as excessive release of cytokines (reviewed in detail by Mangalmurti et al. [8]). Notably, they accompany the acute phases of many respiratory viruses like IAV or SARS-CoV-2 [9]. For example, infection with a highly pathogenic influenza virus elevates levels of IFN-γ, IL-1, IL-6, and TNFα in patients [10,11,12]. The underlying molecular reasons are multiple, ranging from enhanced triggering of viral sensors to metabolic changes induced by IAV [13]. Another notable recent example of pathogenesis related to cytokine storms is caused by SARS-CoV-2, defined by a high abundance of IL-6 but surprisingly low levels of IFNs [14]. However, for both viruses sudden and aberrant positive feedback to immune cells and excessive release of pro-inflammatory cytokines eventually contributes to the severity of the acute symptoms. Notably, the intensity of the cytokine storm in influenza patients was reported to correlate with the severity of the diseases and mortality in COVID-19 has been associated with increased aberrant release of pro-inflammatory cytokines [13,14].

### 1.2. What Are Post-Acute Sequelae of Viral Infections?

**Figure 1:**
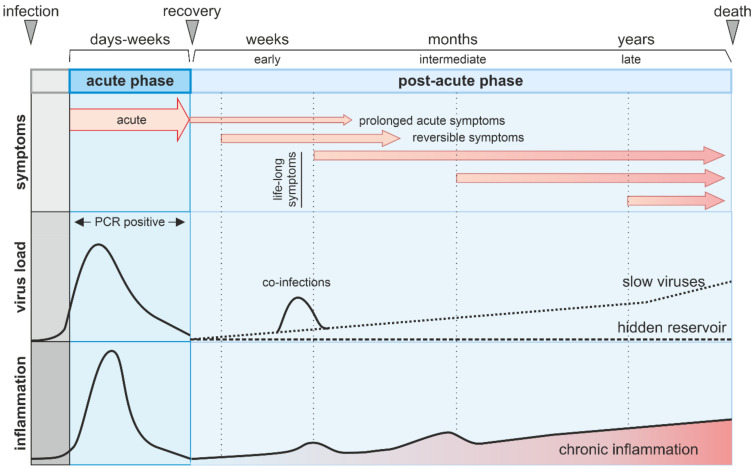
Schematic overview of symptoms, virus load and inflammation levels in the pre-acute, acute and post-acute phase of a (non-persistent) viral infection. After the initial infection and an incubation period (pre-acute phase, gray) a patient enters the acute phase with PCR-detectable virus loads and a peak of inflammation (blue) usually accompanied by acute clinical symptoms. The post-acute phase (light blue), which is usually days to weeks after the beginning of the acute phase is characterized by no viral replication, slow replication or viral components/virions in hidden reservoirs that are undetectable by diagnostic PCR. Different types of symptoms are indicated by arrows. During the post-acute phase inflammation levels may rise again eventually leading to a state of chronic inflammation.

The acute phase of non-persistent viruses is characterized by a virus-specific set of acute symptoms and detectable viral replication (Figure 1). These symptoms usually disappear once the virus becomes undetectable by PCR. Recent research has highlighted that the pathogenesis of non-persistent viruses may extend beyond the acute phase (Figure 1) [15,16]. Post-acute sequelae (PAS) are (often rare) complications and clinical manifestations linked to a viral infection but occurring after the acute phase. However, the exact definition of the post-acute phase and thus post-acute sequelae is currently under debate and differs between virus species. For example, the acute phase of COVID-19 usually lasts two to four weeks, during which the virus is actively shed and the onset of post-acute sequelae is usually considered after week 5 [16]. IAV is usually cleared within 5–6 days in adults and post-acute symptoms may include chronic fatigue that has been reported up to six weeks post the initial infection [17].

PAS are known by different names, for example Post-virus syndrome or long hauler symptoms [18,19]. Alternatively, terms for specific viruses are in use, like “PostPolio Syndrome” for sequelae of Poliovirus infections or “long COVID” and ”Post-Acute Sequelae of COVID-19” (PASC), for sequelae of COVID-19 [20,21,22]. In this review, we generally use the term PAS for symptoms appearing after the acute phase of a viral infection. This includes symptoms from the acute phase that may persist longer than expected (prolonged acute symptoms, Figure 1) and symptoms of a different nature than the acute symptoms that manifest (Figure 1) weeks, months or sometimes even years after the acute infection. While some of the post-acute sequelae may be transient, others are irreversible. In general, symptoms are diverse but often include chronic fatigue, decline in quality of life, neurological symptoms and auto-inflammatory/auto-immune diseases [22,23]. 

Post-virus sequelae are sometimes dismissed as part of the recovery of the body after an infection, but recent research has highlighted specific underlying molecular reasons [16]. Especially aberrant reactions of the innate immune system and subsequent chronic inflammation are proposed to play a key role during PAS of non-persistent viral infections. 

## 2. Post-Acute Sequelae of Non-Persistent Viruses

While it is well-established that persistent viruses cause symptoms beyond their initial acute phase, the concept that infections with non-persistent viruses are responsible for long-term sequelae is relatively recent. The occurrence of post-acute disorders is not limited to certain virus species, but seems to be prevalent across different virus families with clinical symptoms depending on the virus species (Table 1).

### 2.1. DNA Viruses

Post-acute sequelae of non-persistent DNA viruses have seldom been described. For example, parvovirus B19 (PVB19) infections were reported to contribute to the development of chronic, inflammatory and neurological diseases such as myalgic encephalomyelitis/chronic fatigue syndrome (ME/CFS) via immune cell alteration, mitochondrial modulation and autoimmunity [24,25,100]. Notably, after adenovirus (AdV) infections post-acute ocular irritations were observed as well as prolonged respiratory complications [28]. However, it is not known whether these sequelae are associated with adenovirus persistence or transient infections. Additionally, PVB19 and AdV could be detected by PCR in the cardiac tissue of patients with dilated cardiomyopathy, indicating a potential role in the development of postinfectious myocarditis [26].

### 2.2. dsRNA Viruses

It was recently reported that reoviruses such as rotavirus A can trigger celiac disease (CD) in the long run. CD is an autoimmune disorder that is caused by a reaction to gluten, which is found in wheat and other grains. Typical symptoms are chronic diarrhea, malabsorption of nutrients, weight loss and anemia [29]. Mechanistically, reoviruses disrupt the intestinal immune homeostasis, eventually facilitating T cell mediated immunity against dietary antigens. Type I IFN and interferon regulatory factor 1 signaling play a central role by blocking regulatory T cell conversion and promoting helper T cell immunity [32]. In addition, rotaviral infections were proposed to induce diabetes mellitus via auto-antibodies and/or affecting the pancreas [30,31]. Notably, pancreas pathology was TLR3 dependent, which usually detects dsRNA. Vaccination against rotavirus was reported to be safe and may even reduce the risk of developing post-acute sequelae [101,102].

### 2.3. (−) RNA Viruses

Human pathogenic viruses belonging to the order of *Mononegavirales* frequently cause severe acute diseases. However, for many members of this order, post-acute sequelae have been reported as well. Measles virus (MeV) infection can lead to a prolonged immunosuppression of around 2 to 3 years by depletion of B and T lymphocytes, which increases susceptibility to other infections and mortality [33,34,35]. Adding to the direct effects MeV has on lymphocytes, persistence of viral RNA for months after clearance of infectious virus may trigger immune dysregulation [36,37]. Furthermore, infection with MeV can also cause subacute sclerosing panencephalitis (SSPE), a progressive neurological disorder characterized by inflammation of the brain. The underlying cause of SSPE is described as a slow MeV infection of the CNS with little or no production of virions [38]. This was suggested due to defects in the viral genome that render the M protein non-functional [39]. The slowly progressing infection also triggers a constant inflammatory response in the brain. SSPE also seems to have a genetic predisposition, as certain polymorphisms in innate immune genes such as TLR3 or TLR4 are more common in patients with SSPE than healthy controls [103]. Nipah virus (NiV), which is also a paramyxovirus, can cause severe neurological sequelae with very late onset or relapses [40,41,42]. Similarly to measles, it has been shown that NiV is able to persist in the CNS of nonhuman primates and cause encephalitis [104].

Ebola virus disease (EVD) is one of the most fatal infectious diseases, with a case fatality rate of 25% to 90% [48,105]. In addition, survivors often suffer under long-term sequelae, predominantly generalized by symptoms like musculoskeletal pain and fatigue but also ocular and auditory disorders and neurological problems [43,44]. Ebola virus (EBOV) may also cause renal failure as a long-term consequence even after apparent recovery of the patient from the acute infection [45]. Traces of EBOV were reported in immune privileged sites (testes, eyes, CNS) for longer periods and EVD survivors were reported to have increases levels of auto-antibodies against dsDNA and heat shock protein 60 and higher levels of pro-inflammatory cytokines (IL-8 and TNFα), chronic immune activation markers (CCL5 and soluble CD40L) and an altered balance of immune cells [46,47]. However, studies have also reported no association with inflammatory markers [49]. The clinical symptoms of EVD include gastrointestinal symptoms and it has been found that survivors exhibit signs that indicate leakage from the gut, such as higher levels of soluble CD14, intestinal fatty acid binding protein (IFABP) and lipopolysaccharide (LPS)-binding protein (LBP) [46]. Infected nonhuman primates exhibited a loss of gut-associated lymphoid tissue (GALT) as well as breaches in the mucosal barrier of the intestines with bacterial invasion [106]. Notably, it has also been reported for the related Marburg virus (MARV) that survivors suffer from general musculoskeletal sequelae like muscle pain and aches (myalgia) or joint stiffness (arthritis) but also neurological symptoms such as psychosis or eye problems like conjunctivitis after recovery [50,51]. Besides EBOV and MARV, survivors of other hemorrhagic fevers also experience severe prolonged symptoms. For example, infection with Lassa virus (LASV) can cause inflammation in the eye, hearing loss and loss of coordination (ataxia) [52,53,54]. Infection of nonhuman primates revealed severe auto-immune mediated vasculitis in the inner ear. Persistent viral genomes in the smooth muscle cells lining the inflamed arteries were also identified. This indicates that dysregulation of the immune response due to constant triggering by LASV viral antigens is the cause of neurological complications [107].

Negative strand RNA viruses infecting the respiratory tract are suspected of causing long-term sequelae such as impairment of lung function or development of long-term respiratory diseases, especially if infection occurs early in life [55,56]. For example, IAV and respiratory syncytial virus (RSV) infections were reported to contribute to the development of asthma in children [58,59,108]. However, the evidence for RSV is inconclusive in showing a causal association [109]. IAV generally causes rather short, acute infections that can, however, lead to pneumonia and hospitalization, especially in the elderly or immunocompromised [57]. In the latter, single cases of prolonged shedding of virus have been reported [110]. Besides the infectious virus, remnants of viral replication can also be a cause of prolonged immune dysregulation as they continuously trigger antiviral immune responses. Transcriptionally active IAV RNA in sites of previous infection has been found in experimentally infected mice. The mice developed persistent lung damage, inflammation, elevated mucus production and hyper-reactivity. The development and chronification of the lung damage was found to be partly dependent on IL-13, suggesting an ongoing type II immune signaling [108]. Consistent with these findings is the remodeling of lungs of infected mice, in which the appearance of chemosensory cells seems to be involved in type II inflammation circuits and surviving club cells that exhibit sustained expression of proinflammatory cytokines [111,112,113].

### 2.4. (+) ssRNA Viruses

Members of the Flavivirus family are well known to cause neurological complications. While Zika virus (ZIKV) is only characterized by mild acute symptoms, recent outbreaks in French Polynesia (2013) and the American continent (2016) showed severe long-term neurological complications in infants and adults [60,61]. The most prominent neuropathological condition of congenital infection is microcephaly, whereas infection in adults may result in encephalitis, myelitis and Guillain–Barre syndrome (GBS) [62,63,64]. GBS is characterized as a sudden onset weakness of the limbs associated with loss of reflexes and ascending paralysis [114]. In around 20–30% this includes the respiratory muscles and can lead to the death of the patient. GBS is an immune mediated demyelinating or axonal neuropathy, which is caused by aberrant immune reactions targeting nerve antigens with a similarity to pathogen antigens. Co-infections with Dengue virus (DENV) or Chikungunya (CHIKV) virus seem to exacerbate the risk and severity of the symptoms [65]. Recent work suggests that patients with neurological complications have high levels of growth arrest-specific 6 (Gas6), which promotes ZIKV infection and downmodulates the type I IFN response [115]. Another member of the Flavivirus family, DENV, is responsible for 400 million estimated infections per year and 100 million clinical manifestations, a growing global burden [116]. On top of that, a large number of symptomatic patients (50%) suffer under long-term sequelae, mainly fatigue and musculoskeletal problems, but also neurological complications like memory loss or GBS [66,67]. There is an association between the FcγRIIa (FcγRIIa-131HH) gene polymorphism, enhanced autoimmune markers and persistent clinical symptoms [68]. West Nile virus (WNV) is a mosquito-borne flavivirus that leads in 25% of cases to West Nile Fever (WNF) and in less than 1% to a neuro-invasive disease [69]. In some patients WNV can persist in the brain and kidney for longer time periods [70,71]. About 50% of WNF survivors suffer under long-term complications like fatigue, myalgia, memory loss and motor problems. The pathological features of these patients overlap with those of Alzheimer’s disease (AD) and Parkinson’s disease (PD) and are presumably caused by direct neural damage and long-term production of proinflammatory cytokines (e.g., IFN-γ, TNFα, Il-1β) secreted by WNV specific T cells [117,118]. In mice, WNV RNA was found up to 6 months post infection [72]. Other members of the Flavivirus family were also reported to cause PAS [73,74].

Other arboviruses, such as alphaviruses can also cause neurological sequelae. Acute CHIKV infection is mainly characterized by fever, severe joint pain and rash but there are also atypical manifestations affecting different organs like the liver, kidney or brain [119]. After the acute phase, rheumatic symptoms (arthralgia, arthritis) and neurological disorders can persist in subacute or chronic form in a significant fraction of patients for several months or even years [75]. The main reason for chronic rheumatic symptoms seems to be the prolonged existence of viral material in joints and subsequent chronic inflammation [120]. The neurologic manifestations can be explained by neuroinvasion and the resulting direct (cell apoptosis) and indirect (immune activation) damage [76].

The ‘New World’ alphaviruses Venezuelan equine encephalitis virus (VEEV), Eastern equine encephalitis virus (EEEV) and Western equine encephalitis virus (WEEV) can infect humans with variable severity. However, infections can cause long-lasting neuronal sequelae like psychological changes and intellectual disabilities [77]. For WEEV it has been shown in a mouse model that CNS infection results in a neuroinflammatory response promoting neuronal injury, protein aggregation and selective loss of dopaminergic neurons, resembling parkinsonism [121]. PAS were also reported for other alphaviruses like Ross River virus and Sindbis virus [78,79].

PAS are attributed to infection by several members of the *Picornaviridae* family. Enterovirus A71 (EV-A71) and coxsackievirus (CV) A16 can cause the hand, foot and mouth disease, which mainly affects children. Severe CNS involvement during the acute phase can lead to long-term sequelae like ventilatory problems, neurodevelopmental delay and cerebellar dysfunction, which is possibly caused by neuronal damage or a systemic immune response [80,81,82,83]. Furthermore, enteroviruses, especially group B coxsackieviruses, are a major cause of viral myocarditis, an inflammation of the heart muscle [27]. Myocarditis can manifest as an acute or chronic (lasting more than 2 weeks) disease. As enteroviruses can infect and replicate in cardiac tissue, immune responses triggered by the virus via TLR8 were suggested to be the underlying cause [122,123]. Myocarditis is not uniquely found in coxsackieviruses but is associated with other viruses as well, e.g., SARS-CoV-2, parvovirus B19 or AdV.

Post-polio syndrome (PPS) is a post-acute sequelae of Poliovirus (PV) infection that occurs in up to 20–80% of cases [84]. The pathogenesis involves damage to the nervous system typically 15–30 years after the initial infection. Symptoms include decreasing muscular function or acute weakness with pain and fatigue. The underlying reasons are currently not well understood. Notably, it shares many features with chronic fatigue syndrome. Increased plasma levels of TNFα, IL-6 and IL-8 were found in patients suffering from PPS [124]. In the acute phase PV is predominantly sensed by TLR3, initiating an anti-viral response [125]. Notably, several studies have suggested that low-level infection is sustained by either PV1, PV2, or PV3 (not by non-polio enteroviruses) [85,86,87]. Additional evidence has shown that family members of PPS patients do not carry PV genomes, thus confirming that PPS patients are not infectious. However, whether possibly persisting PV has a role in the development of these post-acute symptoms is unclear. In addition, it has been speculated that an autoimmune reaction triggered by the infection may be involved [88].

### 2.5. Post-Acute Sequelae of COVID19 (PASC)

One virus has recently captured the spotlight in research on post-acute sequelae: SARS-CoV-2, the causative agent of COVID-19 [126]. In its acute phase, immune triggering and manipulation by SARS-CoV-2 leads to an atypical cytokine storm and respiratory symptoms in severe cases [7,127]. Also termed long COVID, post-acute sequelae of COVID-19 (PASC) has been reported by patients. Risk factors include old age, excess weight and inflammatory predispositions such as asthma [22]. A recent study reported that about 30% of COVID-19 outpatients or hospitalized patients report persistent symptoms [89]. Among these are fatigue, abnormal thermoregulation, skin diseases, intestinal symptoms up to diabetes leading to significant symptom burden and failure to fully recover and return to previous work levels [22,23,90,91,92,93,94]. Notably, the previously epidemic coronaviruses SARS-CoV-1 and MERS-CoV have induced a similar set of symptoms in their post-acute phase [97,98,99]. Due to the fact that little time has passed since the advent of the pandemic at the beginning of 2020 and the discovery of the causative agent, SARS-CoV-2, symptoms and underlying reasons of PASC are currently emerging. It is evident that in its acute phase SARS-CoV-2 manipulates and imbalances the innate immune responses, and thus aberrant innate immune reactions in the post-acute phase seem likely [7]. Indeed, chronic inflammation has been suggested as being among the underlying causes of PASC [22]. It was suggested that patients developing PASC generally had higher pro-inflammatory cytokine levels, among them TNFα [128]. It has been speculated that there may be a hidden reservoir of SARS-CoV-2 and studies have reported the persistence of viral proteins and RNA in intestinal biopsies [129,130]. Slow replication or traces of viral components could provide triggers for the immune system, fueling chronic inflammation and facilitating PASC [95,129]. The complement system was also implicated with the development of PASC [131,132]. Furthermore, it was noted that during acute COVID-19, autoantibodies, e.g., against the type I IFN system, contribute to the pathogenesis and development of severe diseases [133,134]. Notably, a small set of children developed severe Kawasaki disease-like symptoms, now termed multisystem inflammatory syndrome in children (MIS-C) [95,96]. Symptoms reported included, but were not limited to, persistent fever, hyperinflammation as well as gastrointestinal symptoms, fatigue and muscle pain. These children developed autoreactive antibodies towards e.g., mitogen-activated protein kinase 2 (MAP2K2) or the lupus antigen (La). In adults with COVID-19, increased levels of autoreactive antibodies similar to rheumatoid arthritis have been reported, which could drive the development of PASC [126]. Increased levels of autoantibodies have been found in people with long-term symptoms of COVID-19 months after infection [135,136]. However, the underlying molecular mechanisms of PASC remain elusive and current mechanistic theories are not mutually exclusive.

## 3. Aberrant Innate Immune Activation in Post-Acute Sequelae

Activation of innate immune responses are usually a very effective anti-viral defense system. However, manipulation of these defenses and damage by the infection may lead to aberrant responses characterized by the unchecked release of pro-inflammatory cytokines such as IL-1α, IL-1β, TNFα, IFN-γ, and IL-6 [137]. Eventually slow, long-term inflammation also called chronic inflammation is established (reviewed in detail in Furman et al. and Lawrence et al. [138,139]). The constant immune activation causes tissue damage and premature aging and exhaustion of the immune system but fails to control the infection. Typical symptoms of chronic inflammation include fatigue, fever, pain, and even the manifestation of auto-immune diseases and neurological complications [137].

### 3.1. Innate Immune Responses in Post-Acute Conditions of Persistent Viruses

During persistent viral infections the virus is not cleared but remains in the host. In the chronic phase this persistence causes diseases, whereas in the latent state little to no impact on the host’s health is observed. Notably, PAS of transient viruses often resemble symptoms found in chronic viral infections, characterized by chronic inflammation. To draw parallels with non-persistent viruses we only outline briefly three examples focusing on their interplay with innate immunity: human immunodeficiency virus (HIV-1), hepatitis B virus (HBV) and herpes-simplex-virus 1 and 2 (HSV-1/2, taxonomically known as human alphaherpesvirus 1 and 2).

During the acute phase, HIV-1 is detected by various PRRs, among them the DNA sensor cGas, but is overall a poor inducer of pro-inflammatory responses including IFNs and the infection is not cleared [140,141]. After an initial peak of cytokines, plasma levels of pro-inflammatory cytokines decline. During latency HIV-1 infection is often accompanied by chronic inflammation, even in elite controllers and patients with antiretroviral medication [142,143]. Viral RNAs in the latent phase are thought to be mainly recognized by Toll-like receptors including TLR7/9 and TLR8 and systemic inflammation was reported to be associated with transcript levels of HIV-1 [144,145]. Notably, plasma levels of IFN-α correlate with disease severity in late stages [146]. Besides directly stimulating immune sensors, disruption of tight junctions of mucosal epithelial barriers following an HIV infection leads to dysbiosis and leakage of microbial stimulants, amplifying systemic inflammatory responses [138,147,148,149,150,151,152]. Chronic inflammation was associated with increased mortality and morbidity, increasing the risks for atherothrombosis, cancer, ageing-related diseases and cognitive impairments [147,153,154,155,156].

HBV is sensed upon initial infection by circulating innate immune cells like dendritic cells, but also recognized in their primary target cells in the liver via TLR3 and RIG-I [157]. However, HBV counteracts TLR and RLR signaling, thus anti-viral cytokines are released at low levels and are not sufficient to clear the infection [158,159,160,161,162]. During the initial stages of persistence, replication of HBV is generally very low, and inflammation almost undetectable [163]. However, in late stages, HBV induces chronic inflammation, eventually leading to cirrhosis and hepatocellular carcinoma in about 90% of cases [164,165,166]. It was reported that production of IFN-γ from NK cells further contributes to chronic inflammation [165]. HBV persistence is often characterized by the presence of large amounts of viral proteins, mainly the hepatitis B surface antigen (HBsAg) [167,168]. Subviral particles containing HBsAg may promote innate immune responses in TLR4 positive cells [169]. In addition, tissue damage induced by HBV and infiltrating immune cells cause a release of DAMPs, further activating inflammatory responses. Chronic HBV infected patients express lower levels of IFN signaling/stimulated genes but higher levels of inflammatory cytokines compared to healthy donors [164,170]. During the chronic phase, effective anti-viral responses are still prevented by HBV proteins [171].

Whereas most Herpesvirus infections are latent and cause no apparent damage to the host, accumulating evidence suggests that HSV-1 infection of the brain, in both symptomatic and asymptomatic individuals, could lead to neuronal damage, encephalitis (HSE) and, eventually, neurodegenerative disorders [172]. HSV-1 was proposed as the trigger of late-onset neurological diseases like Alzheimer’s [173,174,175]. Notably, it was shown that activity of the innate immune system protects against neurological complications [176]. It was recently reported that herpes-simplex-virus-2 (HSV-2) patients with defects in the autophagy genes ATG4A and LC3B2 develop recurrent meningitis (Mollaret’s meningitis), a recurrent or chronic form of inflammation of the protective membranes covering the CNS [177,178]. Mechanistically, autophagy mediates the clearance of HSV-2 antigen in non-defective cells. Genetic disruption of *ATG4A* and *LC3B2* expression led to enhanced viral replication and subsequent cell death in patient cells and cell lines [177].

In summary, chronic inflammation is the underlying cause of many symptoms and sequelae of persistent viruses including not only HBV, HIV and Herpesviruses but also for example papillomaviruses and hepatitis C virus [23,179]. Innate immune activation is triggered by the continuous presence of viral components and further fueled by tissue damage and dysbiosis. However, recent reports highlight that activation of parts of the innate immune system have protective effects against PAS of HSV-2.

### 3.2. Chronic Inflammation Induction by Non-Persistent Viruses

**Figure 2:**
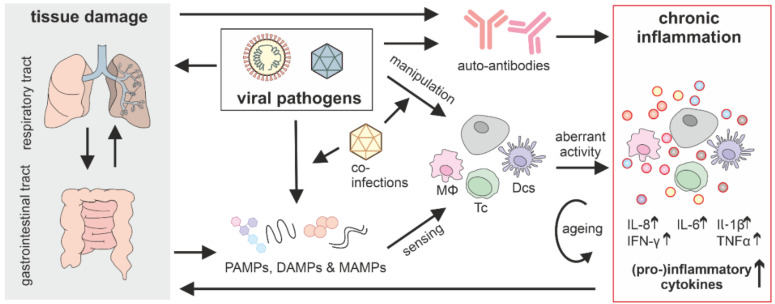
The complex development and amplification of chronic inflammation after an acute viral infection. Viruses trigger the innate immune sensors by PAMPs, but also manipulate the signaling pathways to negate the anti-viral effects of innate immune activation. In addition, many viral pathogens cause tissue damage (grey box), leading to commensal infiltration and further innate immune activation via DAMPs or MAMPs. Dysregulation of immune pathways by viruses, auto-antigen mimicry and tissue damage facilitate the induction of auto-antibodies. Eventually, all these factors cause aberrant activation of immune cells and normal cells leading to (chronic) secretion of inflammatory cytokines. Co-infections further fuel and amplify imbalancing of innate immunity by providing PAMPs but also manipulating cellular signaling cascades. The aberrant activity is further amplified by immune ageing. MΦ, macrophages; Tc, T cells; DCs, dendritic cells; non-immune cells (grey).

Non-persistent viruses generally cause a transient infection that is cleared within days or weeks. Thus, viral replication and gene expression, a key driver of chronic inflammation of persistent viruses, is usually absent in the post-acute phase. However, chronic inflammation was still reported for many non-persistent viruses. Recent years have proposed a few key factors that potentially initiate and drive chronic inflammation: (I) Dysbiosis and tissue damage in the gut and other mucosal surfaces like the respiratory tract, (II) immune exhaustion and ageing, (III) co-infections with other pathogens, (IV) auto-antibodies triggered by the initial infection or (V) continuous triggering and modulation of innate immune responses by viral remnants of the acute phase such as nucleic acids or protein components or slow replication in ‘hidden’ viral reservoirs (Figure 2). Notably, these causes are not mutually exclusive but likely all contribute to the emergence of chronic inflammation in the post-acute phase.

The gut microbiota was proposed to be a major factor in innate (and adaptive) immune processes [180,181]. During homeostasis in healthy individuals the microbiota has important functions ranging from maintaining tolerance towards innocuous antigens to training of important components of both the innate and adaptive immune systems. Due to its pivotal role in maintaining the balance between stimulation and immune responses, imbalance of the gut microbiota has been linked to many subsequent disorders, among them inflammatory diseases, autoinflammatory diseases and diabetes (reviewed in detail by Belkaid et al. and Zheng et al. [180,181]). Upon viral infection, this delicate balance is often disturbed and barrier tissues are affected. In the gastrointestinal tract viral infections may cause dysbiosis, leading to the invasion of bacteria with inflammatory properties. It is thought that the translocating or invading microbiota mainly triggers pattern recognition receptors via MAMPs. Classically, these triggers include bacterial lipopolysaccharides (LPS) that bind to Toll-like receptor 4 (TLR4), eliciting innate immune signaling cascades that eventually induce the expression and secretion of pro-inflammatory cytokines [182]. However, bacterial RNA sequences as well as lipopeptides and unmethylated CpG oligodeoxynucleotide DNA are also detected by various TLRs. Other MAMPs like microbial toxins are detected by intracellular NOD-like receptors leading to inflammasome responses and secretion of IL-1β and IL-18 [183]. Major cell types involved in the detection of invading microbials are resident macrophages and dendritic cells, which upon sensing respond by releasing high levels of TNFα and IL-23 [184]. Eventually detection of the invading commensals leads to inflammatory responses, which may further fuel inflammation by the viral pathogen. Not only in the gut, but also damage to mucosal surfaces in the lung, will lead to detrimental immune activation also in the long run by inducing tissue damage [185]. For example, sustained inflammasome activity and type I IFN release during IAV infection of mice was mediated by inflammatory monocytes, which in turn damaged the mucosal surfaces [186]. Notably, it was proposed that the gut-lung axis of inter-organ communication may shape the inflammatory response especially against respiratory infections [187]. Other mucosal surfaces like the genital tract may be affected in a similar way, but are less well studied [188].

Some studies have suggested that younger people are less at risk from post-acute sequelae [189]. In many inflammatory diseases, immune-ageing and senescent cells contribute to the inflammatory microenvironment [190]. Recently, it has been shown that viral infections, including mouse coronaviruses, trigger inflammatory responses from senescent cells in a mouse model, which contribute to the initial cytokine storms, but may impact post-acute symptoms [191]. If accumulation of senescent cells or immune-ageing are prevented, age-related diseases are delayed [192]. However, the relationship between senescence and viruses is complex and not fully understood yet [193]. Chronic inflammation eventually also leads to premature ageing of the innate immune system, further facilitating dysregulated responses [173,194,195]. Thus, the organism may enter a vicious cycle of inflammation fueled by immune ageing, which in turn causes faster ageing and more aberrant inflammation.

Despite increased levels of inflammatory cytokines and anti-viral gene expression, individuals with chronic inflammation are often more susceptible towards secondary infections [22,138]. In rare cases, infections with non-persistent viruses have even been reported to induce a reset of our immunological memory [33]. It may be tempting to speculate that secondary infections could cause symptoms attributed to PAS. However, secondary infections alone do not explain prolonged symptoms and most PAS often differ from the acute symptoms. These secondary infections may further fuel the state of chronic inflammation, providing additional triggers to immune sensors. Incoming viral proteins may manipulate innate immune pathways, again leading to aberrant reactions, and thus eventually facilitate development of PAS [22,196]. Notably, acute infections often occur in hosts that are (acutely and/or latently) infected with multiple other viruses. Thus, the interplay between an acute virus and the existing virome has to be considered and may shape chronic inflammation and therefore also PAS of non-persistent viruses [197,198].

Disturbance of adaptive and innate immune signaling pathways during an acute infection often results in transient autoimmune responses that in rare cases progresses into an established abnormal recognition of self-antigens, leading to auto-immune diseases [199]. Molecular mimicry in pathogens like viruses, i.e., having similar or structurally related epitopes as endogenous proteins, was suggested to be among the underlying causes [200]. In addition, tissue damage induced by the viral infection leads to the release of normally intracellular components. While predominantly a process of adaptive immunity, activation of the innate immune system by DAMPs at sites of tissue damage facilitates the induction of auto-antibodies. Innate immune activation stimulates antigen-presenting cells and induces migration of immune cells at sites of tissue damage [201,202]. Using a mouse model, it was shown that self-peptides fail to induce auto-antibodies without innate immune stimulation [203]. Furthermore, the triggering of innate immunity during viral infections or by LPS was reported to prevent or delay peripheral tolerance, thus promoting the establishment of auto-immunity [204,205]. Adaptive immune processes that induce inflammation by auto-antibodies are reviewed in detail by Ludwig et al. and Elkon et al. [206,207]. Notably, PAS by a wide range of different viruses are defined by the presence of auto-antibodies. For example, the induction of auto-antibodies against gluten in food after rotavirus A infection may lead to CD. SARS-CoV-2 infected children in rare cases developed systemic inflammation (MIS-C) [95,96] caused by auto-antibodies. Adult COVID-19 patients were also reported to show increased levels of autoreactive antibodies [126,135].

Finally, many classically non-persistent viruses leave traces that linger in the organism long after remission of the acute symptoms. On top of that, many non-persistent viruses were suggested to linger and slowly replicate in a ‘hidden reservoir’ within the human host, escaping classical detection methods and avoiding the induction of acute symptoms. Technically, this would be a form of persistence, blurring the lines between persistent and non-persistent viruses. In the case of MeV-triggered SSPE it is proposed that slow replication might occur in the brain, potentially by defective virus [38]. Other classically non-persistent viruses like rubella virus or rabies virus may also enter a quasi-persistent state by slow virus replication [208,209]. Continued presence of viral RNA was also observed for other RNA viruses like IAV and EBOV [108]. However, in many cases it is unclear whether active viral replication still occurs. Analysis of persistent virus in semen, urine and aqueous samples suggests that persistent EBOV is in a low-level replicative phase, and is not a dormant or latent infection [210,211,212]. The continuous presence of viral components or PAMPs may cause chronic offence to the immune system. Furthermore, low viral gene expression or even replication might in turn antagonize innate immune responses, which may otherwise lead to clearance of the pathogen, resulting in imbalanced innate immunity. Reservoirs often include in tissues that are not the primary targets of the acute infection, like the brain (for MeV) or the bone marrow (for EBOV). One flaw of the current PCR-based diagnostic method is its limitation to easily-accessible tissues/cells. Classically, this includes blood of a patient or swabs from mucosal surfaces. However, viruses or viral components are often hidden in other tissues, such are neuronal tissues, thus evading detection [213,214].

Acute COVID-19 was found to lead to more severe symptoms and higher mortality in males [215,216]. Recent studies highlighted that sex differences, particularly in the immune system, may be a key factor [215]. While male COVID-19 patients had higher levels of pro-inflammatory cytokines in the plasma, female patients displayed a more robust T cell activation [217]. Notably, an increasing number of studies have shown that post-acute sequelae are more often observed in females [189,218]. It has been speculated that either a generally more active immune system or more rapid emergence of auto-antibodies may be among the underlying reasons [23,219,220].

In summary, many non-mutually exclusive factors contribute to chronic inflammation after the acute phase of a non-persistent infection (Figure 2), among them tissue damage, auto-antibodies and continuous exposure of cells to viral PAMPs. Immune ageing and co-infections further exasperate aberrant responses eventually leading to chronic inflammation, characterized by increased presence of pro-inflammatory cytokines.

## 4. Conclusions and Perspectives

Currently, the extent of long-term damage caused by transient viral infections is only beginning to emerge. Retrospectively, many diseases may be associated with or triggered by an initial viral infection. PAS of non-persistent viruses are often strikingly similar to symptoms observed for persistent virus infections, and so might be their underlying molecular mechanisms. Chronic inflammation that fails to clear the infection was identified as one of the key drivers of the pathogenesis of persistent viruses and may have a similar prominent role in PAS of non-persistent viruses. Disruption of mucosal surfaces during an acute infection, allowing commensal bacteria to infiltrate and trigger immune reactions, is certainly one of the drivers of inflammation. Another major factor that induces chronic inflammation are auto-reactive antibodies emerging during the acute infection. Furthermore, emerging evidence showed that many viruses or viral components remain in their hosts longer than expected, either by slowly replicating in ‘hidden’ reservoirs or as components that are not cleared. Secondary infections that provide additional stress for an already imbalanced immune system may further amplify inflammation. Typically, hosts are already infected by one or more persistent viruses and the virus–virus interplay may contribute to aberrant innate immune signaling [196]. Lastly, aberrant inflammation is further promoted by ageing of the immune system.

It should be noted that dysregulation of the innate immune system is certainly an important contributing factor, but likely not the sole cause of PAS. Responses of the adaptive immune system play a central role, as well [221,222]. Factors like the emergence of auto-antibodies, irreversible tissue damage or alterations/ageing in adaptive immune responses that do not directly impact inflammation levels may contribute. Notably, these factors are not mutually exclusive but likely to act synergistically.

Unfortunately, we are often lacking proper in vitro and in vivo models to study molecular details of PAS. This is also due to the fact that it takes a long time after the initial infection of an organism to develop the symptoms. Furthermore, inflammatory responses often involve many different cell types working together on an organismic level. Thus, studies using model organisms may help to define the pathways involved, which may be recapitulated in cell culture models. Some non-persistent viruses such as VSV and AdV are in use as vaccine platforms or delivery vehicles for gene therapy, often as replication incompetent delivery shuttles [223,224]. Thus, long-term consequences are not anticipated but should be monitored in phase IV clinical studies.

Prevention of PAS could be considered in treatment of acute viral infections in the future. Notably, some vaccines were previously reported to reduce PAS of viral infections, underlining the importance of vaccines not only in preventing the acute disease but also long-term damage [101,102]. For PASC, this is currently being debated. Monitoring of inflammation levels after the acute phase could be used as an indicator of potentially developing PAS. A therapeutic approach would thus be to dampen (aberrant) immune reactions, but studies from persistent viruses also remind us that parts of the innate immune system like autophagy may also be required to limit further damage. Thus, re-balancing innate immune activity and decreasing the overall inflammatory burden but allowing effective anti-viral response may very well be one of the key issues in preventing and treating PAS.

Due to the rising public and scientific interest in PAS of viruses in the aftermath of the first months of the COVID-19 pandemic, many clinical and molecular studies have been launched, including research on how vaccines may impact post-acute symptoms [225]. However, drawing parallels with previous insights on PAS of other non-persistent viruses may help us understand how PASC develops and can be treated or prevented. Vice versa, future studies will undoubtedly generally improve our understanding of the common molecular mechanisms of post-acute sequelae of non-persistent viral infections, may provide new models for molecular studies and eventually clarify the contribution of aberrant innate immune activity.

## Figures and Tables

**Table 1:** Overview of non-persistent viruses and post-acute sequelae.

Virus	Genome	Family	PAS	References
PVB19	linear ssDNA	*Parvoviridae*	myalgic encephalomyelitis, chronic fatigue syndrome, myocarditis	[24,25,26,27]
AdV	linear dsDNA	*Adenoviridae*	ocular irritations, respiratory complications, myocarditis	[26,27,28]
Rotavirus A	dsRNA	*Reoviridae*	Celiac disease, diabetes mellitus	[29,30,31,32]
MeV	(−) ssRNA	*Paramyxoviridae*	immunosuppression, immune dysregulation/chronic inflammation, SSPE	[33,34,35,36,37,38,39]
NiV	(−) ssRNA	*Paramyxoviridae*	Neurological sequelae, relapsed encephalitis	[40,41,42]
EBOV	(−) ssRNA	*Filoviridae*	Fatigue, musculoskeletal pain, ocular and auditory disorders, neurological problems, renal failure	[43,44,45,46,47,48,49]
MARV	(−) ssRNA	*Filoviridae*	myalgia, arthritis, conjunctivitis, psychosis	[50,51]
LASV	(−) ssRNA	*Arenaviridae*	Eye inflammation, hearing loss, ataxia	[52,53,54]
IAV	(−) ssRNA	*Orthomyxoviridae*	Asthma, reduced lung function, pneumonia	[55,56,57]
RSV	(−) ssRNA	*Paramyxoviridae*	Asthma, reduced lung function	[55,56,58,59]
ZIKV	(+) ssRNA	*Flaviviridae*	Encephalitis, myelitis, GBS	[60,61,62,63,64,65]
DENV	(+) ssRNA	*Flaviviridae*	Fatigue, musculoskeletal pain, memory loss, GBS	[63,65,66,67,68]
WNV	(+) ssRNA	*Flaviviridae*	Fatigue, myalgia, memory loss, motor problems, neurological problems	[69,70,71,72]
TBEV	(+) ssRNA	*Flaviviridae*	neurological complications, cognitive impairment, tremor, aphasia, sleep disorders, vertigo	[73]
JEV	(+) ssRNA	*Flaviviridae*	intellectual disabilities, neurological sequelae, motor problems, convulsions	[74]
CHIKV	(+) ssRNA	*Togaviridae*	arthralgia, arthritis, neurological disorders	[64,65,75,76]
VEEV	(+) ssRNA	*Togaviridae*	psychological changes and intellectual disabilities	[77]
EEEV	(+) ssRNA	*Togaviridae*	psychological changes and intellectual disabilities	[77]
WEEV	(+) ssRNA	*Togaviridae*	psychological changes and intellectual disabilities	[77]
RRV	(+) ssRNA	*Togaviridae*	arthralgia, fatigue, arthritis, joint problems	[78]
SINV	(+) ssRNA	*Togaviridae*	joint problems, arthritis, rheumatological symptoms, arthralgia	[79]
EV A 71	(+) ssRNA	*Picornaviridae*	ventilatory problems, neurodevelopmental delay, cerebellar dysfunction	[26,27,80,81,82,83]
CV	(+) ssRNA	*Picornaviridae*	ventilatory problems, neurodevelopmental delay, cerebellar dysfunction, myocarditis	[26,27,81]
PV	(+) ssRNA	*Picornaviridae*	decreasing muscular function, acute weakness, pain, fatigue	[84,85,86,87,88]
SARS-CoV-2	(+) ssRNA	*Coronaviridae*	PASC: fatigue, abnormal thermoregulation, skin diseases, intestinal symptoms, diabetes, reduced respiratory capacity MIS-C: persistent fever, hyperinflammation, gastrointestinal symptoms, muscle pain	[22,23,89,90,91,92,93,94,95,96]
SARS-CoV	(+) ssRNA	*Coronaviridae*	fatigue, reduced lung capacity and ventilation, myalgia, mental health problems	[97,98]
MERS-CoV	(+) ssRNA	*Coronaviridae*	Chronic fatigue, mental health problems, reduced lung function	[99]

## Data Availability

Not applicable.
